# Synaptic GluN2B/CaMKII-α Signaling Induces Synapto-Nuclear Transport of ERK and Jacob

**DOI:** 10.3389/fnmol.2016.00066

**Published:** 2016-08-10

**Authors:** Michelle Melgarejo da Rosa, PingAn Yuanxiang, Riccardo Brambilla, Michael R. Kreutz, Anna Karpova

**Affiliations:** ^1^Research Group Neuroplasticity, Leibniz Institute for NeurobiologyMagdeburg, Germany; ^2^Division of Neuroscience, School of Biosciences, Neuroscience and Mental Health Research Institute, Cardiff UniversityCardiff, UK; ^3^Leibniz Group “Dendritic Organelles and Synaptic Function”, Center for Molecular Neurobiology Hamburg (ZMNH), University Medical Center Hamburg-Eppendorf, ZMNHHamburg, Germany

**Keywords:** GluN2B, ERK1/2, Jacob/NSMF, synapse-to-nucleus, CaMKII-α, RasGRF2

## Abstract

A central pathway in synaptic plasticity couples N-Methyl-D-Aspartate-receptor (NMDAR)-signaling to the activation of extracellular signal-regulated kinases (ERKs) cascade. ERK-dependency has been demonstrated for several forms of synaptic plasticity as well as learning and memory and includes local synaptic processes but also long-distance signaling to the nucleus. It is, however, controversial how NMDAR signals are connected to ERK activation in dendritic spines and nuclear import of ERK. The synapto-nuclear messenger Jacob couples NMDAR-dependent Ca^2+^-signaling to CREB-mediated gene expression. Protein transport of Jacob from synapse to nucleus essentially requires activation of GluN2B-containing NMDARs. Subsequent phosphorylation and binding of ERK1/2 to and ERK-dependent phosphorylation of serine 180 in Jacob encodes synaptic but not extrasynaptic NMDAR activation. In this study we show that stimulation of synaptic NMDAR in hippocampal primary neurons and induction of long-term potentiation (LTP) in acute slices results in GluN2B-dependent activation of CaMKII-α and subsequent nuclear import of active ERK and serine 180 phosphorylated Jacob. On the contrary, no evidence was found that either GluN2A-containing NMDAR or RasGRF2 are upstream of ERK activation and nuclear import of Jacob and ERK.

## Introduction

In recent years signaling from synapse-to-nucleus has gained increasing attention and the hypothesis that nuclear gene expression is controlled by a fast Ca^2+^-mediated component and a slower component involving synapto-nuclear protein import has received support from several lines of evidence (Proepper et al., 2007; Jordan and Kreutz, 2009; Karpova et al., 2012; Kaushik et al., 2014; Panayotis et al., 2015). Jacob is one of the proteins that can be transported into the nucleus and that couples NMDAR activity to gene transcription (Dieterich et al., 2008; Kindler et al., 2009; Behnisch et al., 2011; Karpova et al., 2013). Long-distance shuttling of Jacob encodes and transduces the synaptic or extrasynaptic origin of NMDAR signals to the nucleus and we could show that Jacob operates as a mobile hub that docks an NMDAR-derived signalosome to nuclear target sites like CREB (Dieterich et al., 2008; Karpova et al., 2013). In brief, ERK1/2 binding and ERK-dependent phosphorylation of the serine 180 residue in Jacob encodes synaptic but not extrasynaptic NMDAR activation. A stable trimeric complex with proteolytically cleaved fragments of the neurofilament α-internexin is formed which protects Jacob and active ERK1/2 against phosphatase activity during retrograde transport. In the nucleus this signalosome-like complex enhances “plasticity related” and “CREB dependent” gene expression as well as synaptic strength (Karpova et al., 2013). Nuclear import following stimulation of extrasynaptic NMDAR results in sustained de-phosphorylation and transcriptional inactivation of CREB (CREB shut-off), a loss of synaptic contacts, a retraction of dendrites and eventually cell death (Dieterich et al., 2008; Rönicke et al., 2011; Karpova et al., 2013; Gomes et al., 2014).

An open question is how synaptic NMDAR signaling is connected to long-lasting ERK activation and nuclear import of Jacob and ERK. Most NMDAR in the forebrain where Jacob is most abundant (Mikhaylova et al., 2014) are of the GluN2B or GluN2A type. Jacob translocates to the nucleus upon the induction of NMDAR-dependent LTP (Behnisch et al., 2011; Karpova et al., 2013) and pharmacological blockage of GluN2B (Dieterich et al., 2008) but not GluN2A (Dinamarca et al., 2016) containing NMDAR prevents its synapse-to-nucleus trafficking. However, the link between NMDAR-mediated Ca^2+^-influx and ERK1/2 activation as well as translocation of active ERK1/2 to the nucleus has been a matter of debate. Three proteins have been proposed to trigger activation of the hippocampal synaptic Ras-MEK-ERK1/2 pathway. These are two members of the family of calcium-sensitive guanine nucleotide releasing factors, called RasGRF1 and RasGRF2 (Brambilla et al., 1997; Krapivinsky et al., 2003; Li et al., 2006; Shaomin et al., 2006; Jin and Feig, 2010; Fasano and Brambilla, 2011), and CaMKII-α (El Gaamouch et al., 2012). RasGRF1 and CaMKII-α were shown to directly associate with the C-terminal tail of GluN2B containing NMDAR and disruption of the interaction resulted in inactivation of ERK1/2 (Krapivinsky et al., 2003; El Gaamouch et al., 2012). Also several other reports (Chen et al., 2007; Jang et al., 2015) are consistent with the coupling of GluN2B-containing NMDARs to ERK1/2 activation. On the contrary it was proposed that RasGRF2 links the activity of synaptic GluN2A-containing NMDAR to ERK1/2 activation (Li et al., 2006). In addition, pharmacological evidence was provided for the coupling of GluN2A-containing NMDAR to ERK1/2-phosphorylation in hippocampal primary neurons (Gao et al., 2010). Yet another study suggested that the synaptic NMDAR dependent ERK1/2 activation pathway is coupled to both GluN2A and GluN2B (Mulholland et al., 2008) and some reports even suggest that stimulation of GluN2B-containing NMDARs results in ERK1/2 inhibition (Chandler et al., 2001; Sutton and Chandler, 2002; Kim et al., 2005). Moreover, the positive and negative regulation of ERK1/2 by GluN2B-containing NMDAR might depend upon the developmental stage and synapse maturation. For instance it appears that in older neurons RasGRF1 is either no longer coupled to ERK1/2 or it might, by unknown mechanisms, inhibit theses kinases, whereas RasGRF2 mediated activation of ERK1/2 contributes to hippocampal LTP maintenance only in mature animals (Li et al., 2006). GluN2B expression levels are highest during early postnatal development, peaking in the hippocampus and cortex during the third postnatal week and then decline to moderate adult levels (Paoletti et al., 2013). In contrast GluN2A expression is up-regulated during the second postnatal week and is highly abundant in adult brain. Moreover, di- and tri-heteromeric NMDAR have been described and the selectivity of antagonists for these different NMDAR is relatively poor (Paoletti et al., 2013). Given this conundrum of conflicting data we decided to address in more detail how synaptic NMDAR are coupled to nuclear translocation of ERK1/2 and Jacob.

## Materials and methods

### Primary neuronal culture, pharmacological treatment, antibodies, and plasmids

Hippocampal and cortical rat (from E18–E19) primary neuronal cultures were prepared as described previously (Dieterich et al., 2008; Behnisch et al., 2011) and all procedures were conducted under established standards of the German federal state of Saxony-Anhalt, Germany, in accordance with the European Communities Council Directive 2010/63/EU on the protection of animals used for scientific purposes. Neurons were cultured in Neurobasal medium (NB, GIBCO/Life Technologies) supplemented with B27 (GIBCO/Life Technologies), L-Glutamine (GIBCO/Life Technologies) and penicillin/streptomycin (PAA Laboratories, Pasching, Austria) till day *in vitro* (DIV) 16 and DIV23. Before fixation neurons at both developmental stages underwent pharmacological treatments as depicted in **Figure 3C**. Briefly, to block spontaneous neuronal activity cells were pre-incubated for 1 h with tetrodotoxin (TTX, 1μM, Alamone labs) defined as control conditions or with TTX followed by wash out and addition of 4-Aminopyridine (4-AP, 2.5 mM, Sigma-Aldrich) and bicuculline (50 μM, bicuculline methiodide, Tocris bioscience) for 30 min to enhance synaptic activity—conditions defined as “synaptic stimulation”. For blocking CaMKII-α activity KN-93 (5 μM, Tocris bioscience) was added directly into the NB neuronal culture media 30 min prior synaptic stimulation and kept for 1 h in total. Ro25-6981 ([R-(R,S)-α-(4-hydroxyphenyl)-β-methyl-4-(phenylmethyl)-1-piperidine propranol], 5 μM) and ifenprodil (5 μM) was obtained from Tocris bioscience, MEK1/2 antagonist U0126 (10 μM) from Cell signaling and NVP-AAM077 ([(R)-[(S)-1-(4-bromo-phenyl)-ethylamino]-(2,3-dioxo-1,2,3,4-tetrahydro-quinoxalin-5-yl)-methyl]phosphonic acid, 50 nM) and anisomycin (7.5 μM) from Sigma-Aldrich. After pharmacological treatments neurons were fixed in 4% formaldehyde (PFA) in phosphate-buffered saline (PBS), permeabilized in 0.1% TritonX-100 in PBS for 10 min, incubated in blocking buffer containing 2% glycine, 2% bovine serum albumin fraction V (ROTH), 0.2% gelatine, 50 mM NH_4_Cl in 1xPBS for 40 min processed for immunocytochemistry (ICC). Primary antibodies were added overnight followed by three subsequent 10 min rinses with PBS before incubation with secondary antibodies. Primary antibody incubation was carried out in humidified chambers at 4°C in 70 μl blocking solution. For Jacob protein detection custom-made anti-panJacob antibodies generated against rat Jacob peptide (aa: *EQPPLPEASGRHKKLER*; Spilker et al., 2016) as well as an anti-pS180-Jacob antibody directed against a phosphorylated rat Jacob peptide (aa: *LVPGPSpPRAFG*; Karpova et al., 2013) were used. Anti-panERK1/2 antibodies were obtained from Cell signaling, anti-pERK1/2 and anti-MAP2 from Sigma-Aldrich, anti-bassoon from Synaptic Systems. Nuclei were stained with 4′,6-diamidino-2-phenylindole (DAPI, Vector Laboratories Inc./Enzo Life Sciences, Lörrach, Germany) for 15 min followed by a wash with PBS and coverslips were mounted with Mowiol 4–88 embedding media (Calbiochem/Merck Chemicals Ltd., Nottingham, United Kingdom).

GFP tagged rat Jacob (WT-Jacob-GFP) was described previously (Dieterich et al., 2008; Karpova et al., 2013). WT-Jacob-tagRFP was generated by exchange GFP with tagRFP (Evrogen) in pEGFP-N1 vector backbone (Clontech). Rat GFP-CaMKII-α was obtained from Addgene (#21226, Shen et al., 1998). Rat tagRFP-CaMKII-α was generated employing PCR followed by homologous recombination approach using Cold Fusion cloning kit (SBI System Biosciences) with the following primers: fw-5′C TCGAGCTCAAGCTTCGAATTCTATGGCTACCATCACCTGCACC 3′ and rev.5′ CTAGATCCGGTGGATCCCCGCCCGGGTCAAGTGGGCAGGACG G3′. Briefly, ptagRFP-C1 vector (Evrogen) was linearized with EcoR1 and recombined with PCR product amplified using Addgene #21226 plasmid as a template. N-terminally tagged FLAG-RasGRF2 and c-terminally tagged RasGRF1-HA constructs were created by subcloning of rat RasGRF2 and rat RasGRF1 into pcDNA-CMV-FLAG and pcDNA3-CMV-HA vectors, respectively. The n-terminal deletion mutant of rat GluN2B NMDAR subunit (GluN2B-840-1482-tagRFP) was generated by PCR and inserted into ptagRFP-N1 vector (Evrogen).

Ras-dead human RasGRF2-R1140A and shRNA2 plasmids were kindly provided by Kimberley F. Tolias (Schwechter et al., 2013). Rat RasGRF2-GFP and human Ras-dead hRasGRF2-R1140A-tagRFP were generated by PCR followed by homologous recombination with the following primers: forward 5′ CAGATCTCGAGCTCAAGTTCCACCATGCAGAAGAGCCTGCGC 3′ and reverse 5′ CGGTGGATCCCGGGCCCG CGGAGCAGGGAGTCGAGGTTC 3′.

### Heterologous co-immunoprecipitation assay

HEK293T cells were co-transfected with plasmids encoding GFP tagged Jacob, CaMKII-α and control GFP together with the c-terminal tail of GluN2B (GluN2B-840-1482-tagRFP), WT-Jacob-GFP with tagRFP-CaMKII-α as well as GFP-CaMKII-α with WT-Jacob-tagRFP using calcium phosphate. At 24 h post-transfection, cells were harvested in RIPA buffer (10 mM Tris–HCl; pH 7.4), 150 mM NaCl, 1% sodium deoxycholate, 0.1% sodium dodecyl sulfate, 1% Triton X-100,) supplemented with protease inhibitor cocktail (ROCHE). Equal amounts (1 ml) of the total cell extracts were incubated with 30 μl of anti-GFP MicroBeads (MACSMolecular) for 1 h and the immune complex was analyzed by immunoblotting. The co-immunoprecipitated cytoplasmic tail of GluN2B and CaMKII-α were detected by western blot (WB) analysis using anti-tagRFP antibodies (Evrogen). Additionally, in reciprocal Co-IPs GFP tagged CaMKII-α was immunoprecipitated with beads and WT-Jacob-tagRFP was detected.

### Acute hippocampal slice preparation, chemical LTP, and extraction of CA1 nuclei

Hippocampi from 4 to 5 weeks old rats were dissected from the brain and acute 350 μm thick slices were prepared using a McILWAIN tissue chopper (Mickle laboratory engineering Co. LTD., Gomshall, UK) in cold saturated with 95%O_2_–5%CO_2_ artificial cerebrospinal fluid (ACSF, 4°C) containing: 110 mM NaCl, 2.5 mM KCl, 1.5 mM MgSO_4_·2H_2_O, 2.5 mM CaCl_2_, 1.24 mM KH_2_PO_4_, 27.4 mM NaHCO_3_, and 10 mM D-glucose (pH 7.3). Prior to stimulation hippocampal slices were incubated in carbogenated ACSF at 31 ± 1°C on submerged grids floating in the 15 ml glass beaker for 1 h. Then they were transferred into new beakers containing rolipram (100 nM, Sigma-Aldrich), forskolin (50 μM, Calbiochem), and picrotoxin (100 μM, Calbiochem; Otmakhov et al., 2004) dissolved in ACSF lacking Mg^2+^ at 31 ± 1°C for induction of chemical LTP (cLTP) in the presence or absence of CaMKII-α inhibitor KN-93 (5 μM). Control slices were incubated in the same buffer without stimulation. After cLTP treatment slices were placed back into original ACSF containing 1.5 mM MgSO_4_·2H_2_O for additional 30 min. Total incubation time with KN-93 was 1 h. Potentiated and control slices were shock frozen and underwent dissection of CA1 regions. Five dissected CA1 regions from the same experimental preparation were pooled together, homogenized and underwent nuclear isolation as described previously (Karpova et al., 2013; Yuanxiang et al., 2014). Briefly, CA1 regions were homogenized in 50 μl of cold (4°C) hypotonic lysis buffer containing 10 mM HEPES, 1.5 mM MgCl_2_, 10 mM KCl (pH 7.9), protease (Complete, ROCHE) and phosphatase (ROCHE) inhibitors. The lysates were centrifuged for 1 min at 11,000 rpm and the pellet fraction containing neuronal nuclei was isolated and re-suspended in 60 μl of lysis buffer. Thereafter homogenate and nuclear fraction were solubilized in 4xSDS loading dye, total protein concentration was measured and 10 μg from each sample was subjected into SDS-PAGE with subsequent immunoblotting for pan-Jacob, panERK/pERK. NeuN (Millipore) and β-actin (Sigma-Aldrich) were used as loading controls for nuclear fraction and homogenate respectively and normalization for loading was included into quantification of pan-Jacob, panERK/pERK nuclear immunoreactivity (IR). Quantitative WB analysis was performed using ImageJ software (Gel Analyzer plug-in) where protein of interest bands intensities were measured and normalized to NeuN and β-actin accordingly.

### Induction of late LTP and immunohistochemistry

Late form of LTP was induced in the stratum radiatum of acute rat hippocampal slices by stimulation of Schaffer-collateral fibers by strong tetanisation (STET) protocol consisting of three 1s trains at 100 Hz (Karpova et al., 2006, 2013; Cai et al., 2010) given 5 min apart in the presence of KN-93 (5 μM) or DMSO accordingly and field excitatory postsynaptic potentials (fEPSPs) were evoked with biphasic rectangular current pulses (200 μs/polarity) in a range of 3–4V. Responses to test stimuli were measured every 3 min throughout the experiment. For detection and quantification of pJacob-S180 nuclear immunoreactivity after high frequency stimulation we utilized published protocols for immunohistochemical stainings (IHC/Karpova et al., 2013). One hour after the first HFS train slices were fixed in 4% PFA in 1x PBS overnight, transferred into 30% sucrose in PBS for additional 24 h. Thereafter slices were cut frozen on a cryostat (Leica CM3050S, Leica biosystems) at 40 μm sections and collected in 1x PBS (pH = 7.4). Sections were washed three times in 1x PBS prior to being immersed into 1xPBS containing 0.2% TrX-100 for 1.5 h on shaker at RT. Two hours of blocking in blocking solution containing 0.1% TritonX100 followed 72 h incubation with anti-pS180-Jacob and anti-MAP2 primary antibodies at 4°C on a shaker. Secondary antibodies were applied in blocking solution for 4 h, washed in 1x PBS. Nuclei were stained with DAPI.

### Validation of shRNA mediated RasGRF2 knock-down, transfections, lentiviral particle production, viral transduction of cortical neurons

Short-hairpin RNA (shRNA) mediated knock-down (KD) approach (LV-shRas-GRF2/see also Bido et al., 2015) was used to downregulate endogenous RasGRF2 protein level in rat hippocampal and cortical primary neurons (targeting sequence and transduction efficiency of the cortical culture are indicated in the Figures S2A and S2E respectively). To validate RasGRF2 plasmid knock-down HEK293T cells grown in 6x well plates in Dulbecco's Modified Eagle Medium (DMEM, GIBCO/Life Technologies) supplemented with penicillin/streptomycin and 10% fetal bovine serum were co-transfected with plasmid expressing FLAG-tagged RasGRF2 or with HA-tagged rat RasGRF1 together with either shRNA1 or a related plasmid control expressing GFP in the ratio of 1:12 (0.3 μg plasmid DNA of FLAG-Ras-GRF2 and 3.7 μg shRNA1 or plasmid control) using calcium phosphate. For validation of down-regulation of rat RasGRF2, but not the human RasGEF-dead-R1140A-RasGRF2 by shRNA2 HEK293T cells were co-transfected as indicated in the Figure S2F plasmid ratios. 48 h post-transfection cells were lysed with 50 mM Tris-Cl [pH 8.5], 150 mM NaCl, 1% NP-40, 0.5% Na-deoxycholate, 0.1% SDS, supplemented with complete protease inhibitor. Equal amount of protein extracts were analyzed by SDS-PAGE and subsequent WB. Protein bands were detected with tag-specific antibodies.

For ICC experiments hippocampal neurons were transfected either with shRNA1 alone or with shRNA2 together with shRNA2 resistant human RasGEF-dead-R1140A-RasGRF2 (Schwechter et al., 2013) to rescue Rac-GEF, but not Ras-GEF RasGRF2 function or plasmid control at DIV17 using Lipofectamine 2000 transfection reagent (Invitrogen) according to the protocol described by Kapitein et al. (2010) and kept for additional 5 days.

Lentiviral particles (shRNA1) for downregulation of endogenous RasGRF2 protein were prepared as described previously (Bido et al., 2015) and used to infect cortical neurons grown in 6x well plates at DIV10/17. Five days after viral transduction neurons were stimulated for 30 min with 4-AP and bicuculline and subsequently lysed with 50 mM Tris-Cl [pH 8.5], 150 mM NaCl, 1% NP-40, 0.5% Na-deoxycholade, 0.1% SDS, supplemented with complete protease inhibitor. The protein concentration was measured by BCA assay and equal protein amounts were analyzed by SDS-PAGE and subsequent WB. Anti-pERK (Sigma-Aldrich) antibodies were used to detect activated endogenous ERK1/2.

### Imaging and analysis

For quantitative ICC and IHC SP5 CLSM system (Leica-Microsystems, Mannheim, Germany) equipped with Diode (405 nm), Argon (458, 476, 488, 496, 514 nm laser lines), Diode Pumped Solid State (DPSS, 561 nm) and HeNe (633 nm) was used. Images were taken with Plan Apo 63x oil NA 1.4 and HCX APO L20x water NA1.0 (Leica) objective lenses. Hippocampal neurons for quantitative ICC analysis were selected randomly based on anti-MAP2 antibodies neuronal specific labeling. In order to cover the nuclear volume of imaged neurons along the z-axis usually 5–9 optical sections with focus depth of 300 nm were scanned in a 512 × 512 pixel format at 8 bit image depth with two times frame average at 400 Hz laser frequency. Images of neurons from experimental conditions within the group were acquired sequentially with the constant laser and detector settings. Subsequently, image analysis was carried out with the ImageJ software (NIH, http://rsb.info.nih.gov/ij/) where nuclear IRs were quantified as measure of pixel intensity value within the region of interest (ROI) as described previously (Dieterich et al., 2008; Behnisch et al., 2011; Karpova et al., 2013). Particularly, the average intensity images for each channel were created from three optical sections within z-projection and were corresponding to the maximum diameter of the nucleus. The thresholding of DAPI staining was used for defining nuclear ROIs that were subsequently applied for the corresponding panJacob, pJacob, panERK, and pERK staining and nuclear IR was calculated as a *mean gray values* of pixel intensity in arbitrary units. Original pixel intensities from 0 to 255 are represented as a gradient lookup table in corresponding figures. Intensity values of panJacob, pJacob, pan ERK, and pERK nuclear IR within experimental group were normalized and described as percentage deviations from the average of the TTX treated controls. Normalized values from at least three independent experiments were pulled together and one-way ANOVA followed by Bonferroni *post-hoc* test was performed. Data in all graphs are represented as mean ±SEM. To detect the level of nuclear pJacob in 40 μm hippocampal sections after LTP induction in the presence or absence of KN-93, CA1 area were imaged with constant laser/detector settings along the z-axis with 400 nm z-step and 25–35 focal sections obtained from the middle part of the slice in order to avoid the surface staining artifact (**Figure 5C**, upper panel). FiJi/ImageJ software was used to calculate maximum intensity projection from four optical sections for each channel. All nuclei detected by thresholding of DAPI and related to MAP2 staining in stratum radiatum were selected for analysis. From maximum intensity projection the mean gray value of pJacob IR was calculated and values from KN-93 treated group were described as percentage deviations from the average of the LTP without KN-93 treatment.

Dendritic spine analysis was performed as described previously (Spilker et al., 2016). Hippocampal neurons transfected at DIV18 either with shRNA1, shRNA2 (RasGRF2 KD), or correspondent control plasmid co-expressing GFP as a volume marker were fixed at DIV23 and proceeded for ICC with anti-bassoon antibodies as a presynaptic marker. For the quantitative analysis of spine density 1–2 dendritic segments per neurons overexpressing GFP extended from the soma for at least 20 μm were scanned with 1024 × 512 resolution and 170 nm z-step size. Maximum intensities projection was calculated from the image stack and synapses were analyzed using FiJi software by identification of co-localizing puncta representing presynaptic marker bassoon by merging with the GFP channel of the images acquired. Spines were counted for 30 μm length of dendrites. For statistical analysis GraphPad Prism 6 software (GraphPad Software, Inc; one-way ANOVA followed by Bonferroni *post-hoc* test) was used.

## Results

### The nuclear translocation of pERK requires activation of synaptic GluN2B-receptors

**Figure 1:**
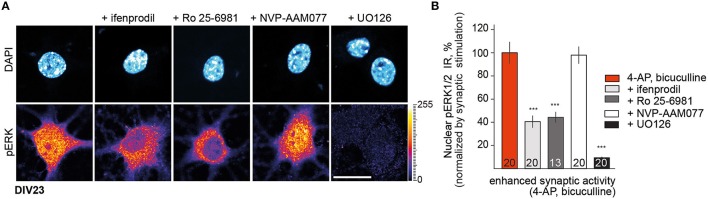
**GluN2B rather than GluN2A receptor activity controls nuclear pERK1/2 level in mature DIV23 hippocampal primary neurons**. **(A,B)** Depicted are representative confocal images of hippocampal neurons treated with ifenprodil (5 μM), Ro-25-6981 (5 μM), NVP-AAM077 (50 nM), and UO126 (10 μM) as a control for pERK activation for 30 min in conditions of enhanced synaptic activity. “*n*-number” depicted in the panels here and below refers to number of neurons from at least three independent experiments. Scale bar is 20 μm. Data represented as mean ± S.E.M.; ^***^p < 0.001; one-way ANOVA followed by Bonferroni *post-hoc* test.

In previous work we have shown that Jacob associates with GluN2B NMDAR complexes and that the protein translocates to the nucleus following stimulation of synaptic and extrasynaptic GluN2B containing NMDAR (Dieterich et al., 2008; Behnisch et al., 2011; Karpova et al., 2013; Dinamarca et al., 2016). Following activation of synaptic NMDAR a stable trimeric complex with the neurofilament α-internexin is formed which protects Jacob phosphorylated at the serine 180 and phosphorylated ERK (pERK) against phosphatase activity during retrograde transport to the nucleus. Removal of inhibitory tone with the GABAa-receptor antagonist bicuculline and blocking of potassium channels with 4-Aminopyridine (4-AP) induces burst firing of excitatory synapses. In agreement to previous findings we observed that incubation of DIV23 hippocampal primary neurons with the GluN2B-antagonists ifenprodil (5 μM) and Ro 25–6981 (5 μM) also resulted in a significant attenuation in nuclear pERK1/2 immunofluorescence upon stimulation of synaptic NMDAR with bath application of 4-AP/bicuculline for 30 min (Figures 1A,B). In contrast, the GluN2A antagonist NVP-AAM077 (50 nM) at low concentrations, that have reportedly little effect on GluN2B-containing NMDAR and that mainly target di-heteromeric GluN2A NMDAR (Foster et al., 2010), had no effect on nuclear pERK levels in response to enhanced synaptic activity. Similarly, nuclear import of pERK1/2 (Figures 1A,B) and Jacob (Dinamarca et al., 2016) was blocked in the presence of ifenprodil but not of NVP-AAM077, indicating that phosphorylated ERK and panJacob are indeed transported to the nucleus in a GluN2B dependent manner. In control experiments we found no nuclear accumulation of pERK when we co-applied the MEK-inhibitor UO126 to the stimulation buffer (Figure 1B).

### Nuclear translocation of Jacob following synaptic NMDAR stimulation in hippocampal primary neurons requires CaMKII-α signaling

**Figure 2:**
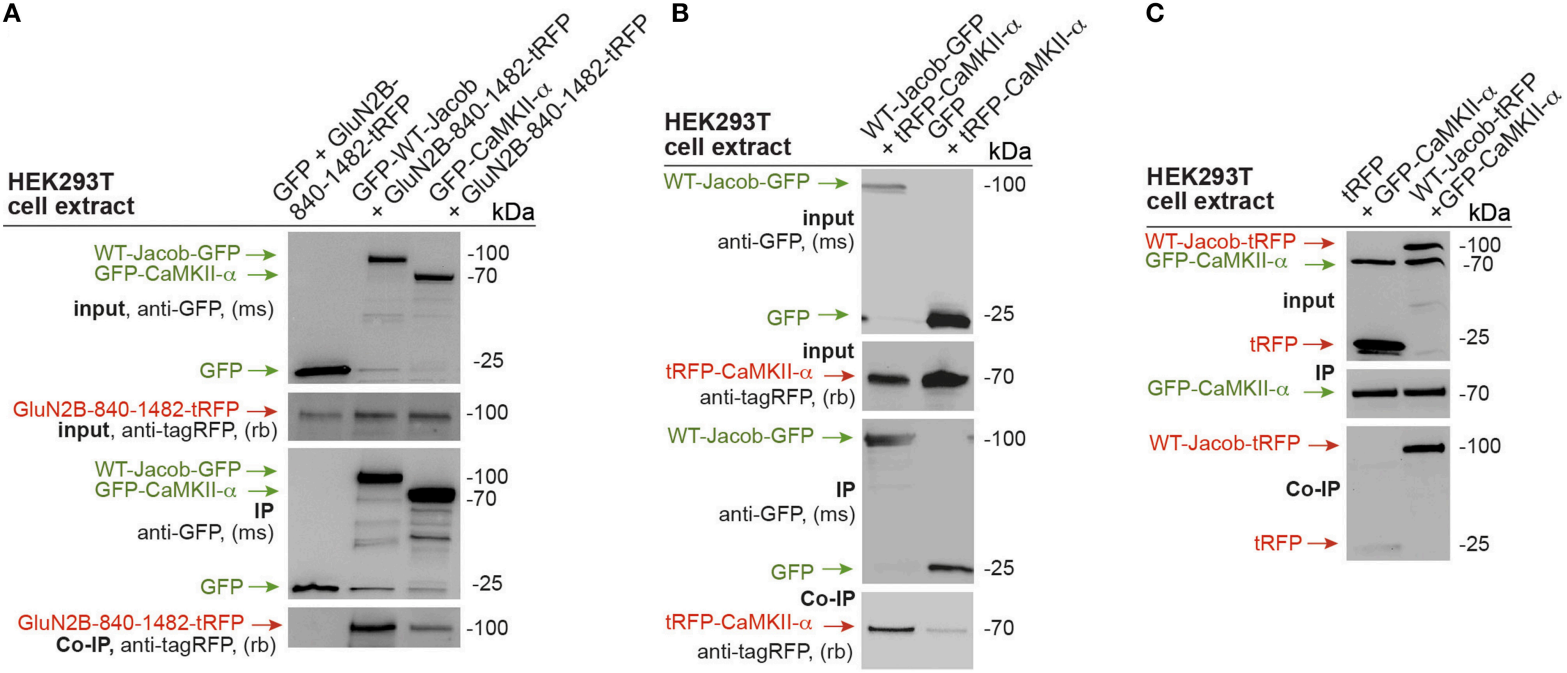
**GluN2B, Jacob, and CaMKII-α are parts of one protein complex. (A)** GFP tagged CaMKII-α and WT-Jacob-GFP but not GFP control co-precipitate c-terminal tail of GluN2B (GluN2B-840-1482-tRFP). **(B,C)** Heterologous co-immunoprecipitation experiments indicate that WT-Jacob and CaMKII-α are parts of one protein complex.

The data suggest that GluN2B- and not GluN2A-containing NMDAR control nuclear level of pERK. Since Jacob following stimulation of synaptic NMDAR requires binding of pERK for nuclear import (Karpova et al., 2013) and Jacob gene knock-out reduces NMDAR-dependent nuclear import of ERK (Spilker et al., 2016), we asked next how activation of ERK is mechanistically linked to NMDARs. Previous co-immunoprecipitation experiments from rat brain lysates suggest that endogenous GluN2B, Jacob, ERK can be part of one complex *in vivo* (Karpova et al., 2013; Dinamarca et al., 2016). Based on these finding we therefore wanted to prove whether an association of Jacob and CaMKII-α exists with the cytoplasmic tail of GluN2B. To this end we transfected HEK-293T cells with tagged constructs and performed heterologous co-immunoprecipitation experiments with GFP-antibody. With these experiments we confirmed an association of rat GFP tagged CaMKII-α as well as GFP-tagged Jacob with the cytoplasmic tail of the GluN2B subunit (GluN2B-840-1482-tagRFP; Figure 2A). No immunoprecipitation was seen in control experiments with lysates from cells transfected with GFP alone (Figure 2A). Interestingly enough, Jacob also appears to be in complex with CaMKII-α (tagRFP-CaMKII-α.) Strong CaMKII-α immunoreactivity was found in the co-precipitate following immunoprecipitation of WT-Jacob-GFP with a tag-specific antibody under stringent conditions (Figure 2B) but not in GFP control. Reciprocal co-immunoprecipitation, where we switched the tag between Jacob and CaMKII-α, yielded similar results (Figure 2C). Thus, it is plausible that GluN2B, Jacob, ERK, and CaMKII-α can be in close proximity to each other at synaptic sites and that CaMKII-α might be upstream of long-lasting ERK activation and subsequent nuclear import of pJacob/pERK.

**Figure 3:**
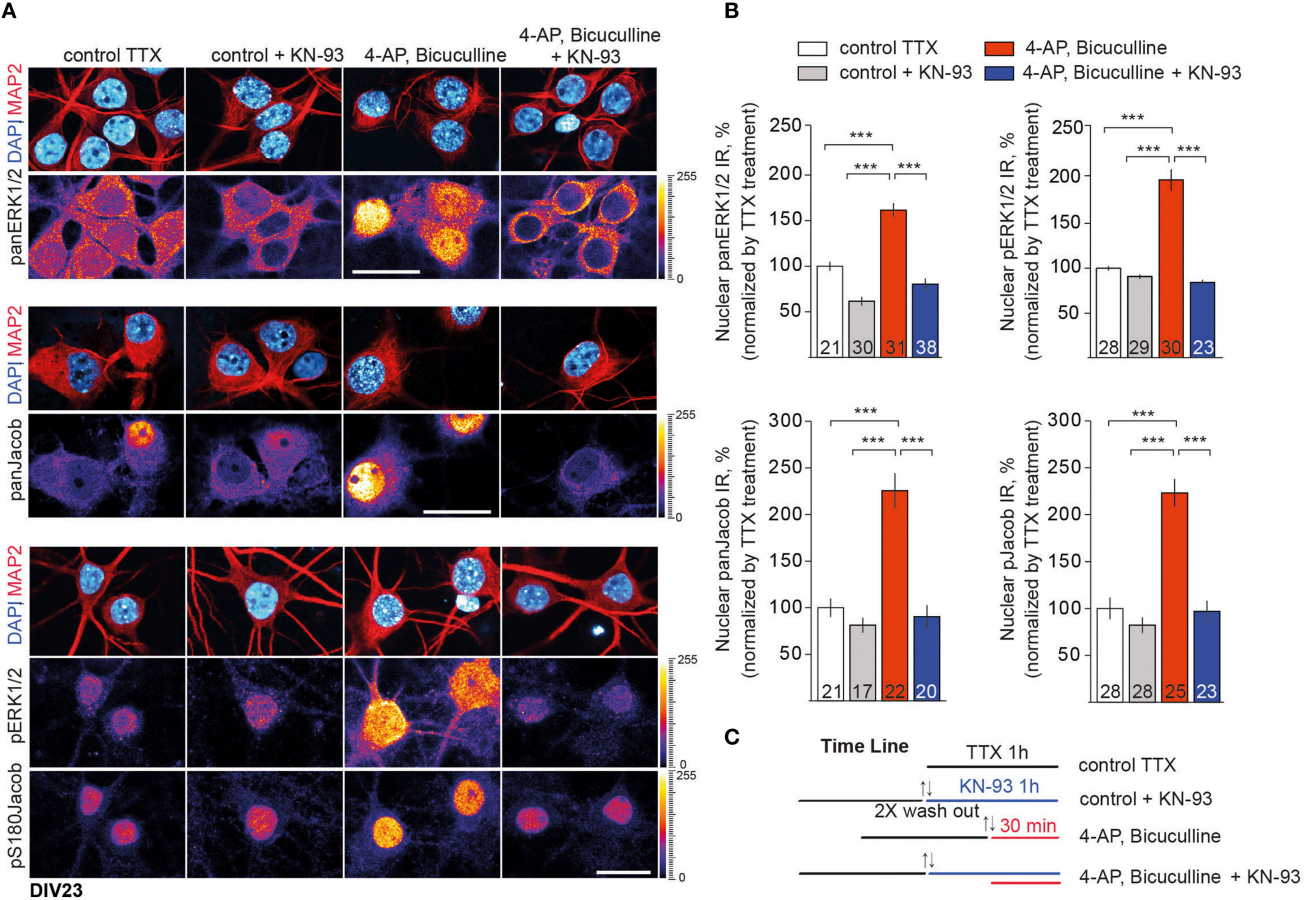
**CaMKII-α activity downstream of GluN2B controls nuclear import of ERK1/2 and Jacob. (A)** Depicted are representative confocal images of DIV23 hippocampal neurons processed for quantitative immunocytochemistry with anti-ERK/pERK and anti-Jacob/pJacob antibodies. Neuronal nuclear ROIs were identified by DAPI matched with MAP2 stainings and nuclear panERK/pERK as well as panJacob/pJacob IR levels were quantified as averaged intensity projection from three optical sections using ImageJ software. Scale bar is 20 μm. **(B)** Data represented as mean ± S.E.M.; ^***^p < 0.001; one-way ANOVA followed by Bonferroni *post-hoc* test. **(C)** Indicated is the time line of the stimulation paradigm.

We next employed the CaMKII-α inhibitor KN-93 to test this hypothesis and performed quantitative ICC analysis of panERK and panJacob as well as of pERK/pJacob nuclear immunofluorescence upon stimulation of synaptic NMDAR in hippocampal primary neurons at DIV16 and DIV23 (Figure S1 and Figure 3C). We used 5 μM of KN-93 since the compound at this concentration has a profound effect on CaMKII-α activity in dendritic spines, whereas inhibition of nuclear CaMKIV and impact on pCREB level is negligible (Redondo et al., 2010). All experiments were performed in the presence of anisomycin in order to exclude an effect of *de-novo* protein synthesis on nuclear ERK and Jacob protein levels. In support of previous work which indicated that CaMKII-α is critically involved in GluN2B-dependent activation of ERK1/2 through a direct interaction with GluN2B (El Gaamouch et al., 2012) we found that the increase in pERK and pJacob nuclear IR in response to enhanced synaptic activity was abolished in the presence of the CaMKII-α inhibitor (Figures 3A,B). Interestingly, panJacob and panERK1/2 immunofluorescence in the nucleus was also reduced, indicating again that nuclear transport of both proteins rather than phosphorylation in the nucleus is controlled by CaMKII-α activity at synaptic sites.

**Figure 4:**
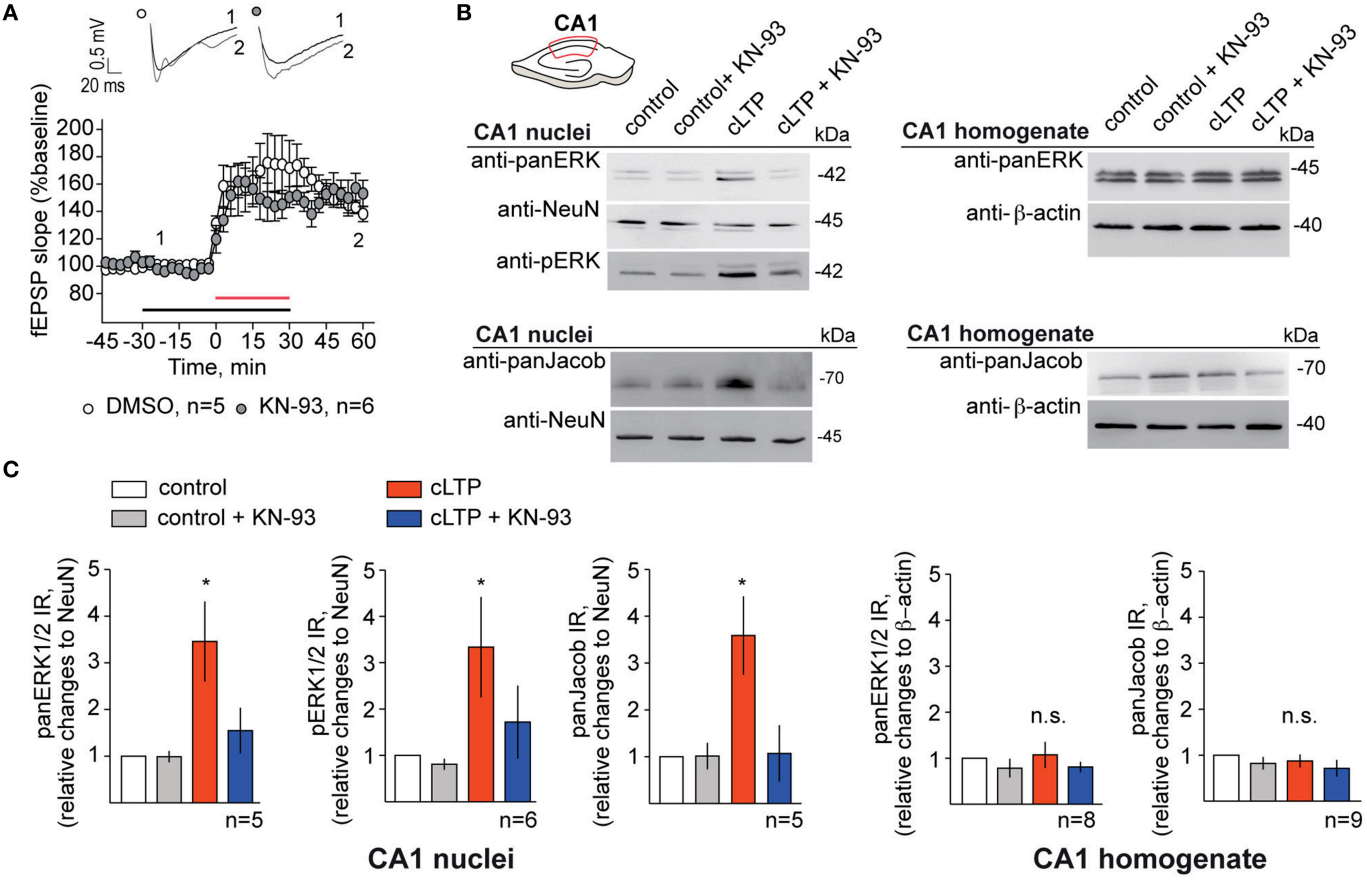
**Blocking of CaMKII-α activity abolishes ERK/Jacob nuclear translocation upon LTP inducing stimuli. (A)** KN-93 in concentration of 5 μM has no effect on cLTP induction and does not decrease the magnitude of LTP in hippocampal slices. Shown are averages of normalized to baseline changes in lateral field EPSP slope for experiments with cLTP in DMSO control ACSF (opened circles; *n* = 5) and in the presence of KN-93 (filled circles; *n* = 6). The red horizontal bar indicates the time line for cLTP, the black bar—the time of KN-93 application. The inset shows representative fEPSPs traces for the time points indicated. Traces recorded during the baseline are indicated in black, whereas traces 60 min after cLTP induction are indicated in gray. **(B)** Induction of cLTP in acute rat slices from 4 to 5 weeks old animals in the presence of KN-93 results in reduction of nuclear panERK/pERK and panJacob in CA1 pyramidal neurons. Representative western blots of nuclear extracts from dissected CA1 areas from five slices incubated at the same conditions as well as of CA1 total lysates are indicated. *n*-number refers to the number of independent experiments **(C)** Results of quantitative WB analysis indicating fold increase in panERK/pERK and panJacob levels in CA1 nuclei and CA1 homogenates. Error bars indicate S.E.M.; ^*^p < 0.05.

We next set out to confirm these results in acute hippocampal slices where we induced NMDAR-dependent chemical LTP (Otmakhov et al., 2004; Boehm et al., 2006), dissected the CA1 area of the hippocampus, extracted the nuclei and performed quantitative immunoblot analysis of nuclear panERK/panJacob as well as pERK levels. Equal loading of nuclear proteins was controlled with an antibody directed against NeuN (for protocol see Yuanxiang et al., 2014). Application of KN-93 had no effect on the induction and maintenance of LTP within the first hour following stimulation (Figure 4A). Protein levels of ERK and Jacob were not affected by chemical LTP induction in CA1 (Figures 4B,C). Likewise to primary neurons we found that bath application of KN-93 prevented LTP-induced nuclear import of Jacob/ERK and accordingly no increase in nuclear pERK was visible (Figures 4B,C).

**Figure 5:**
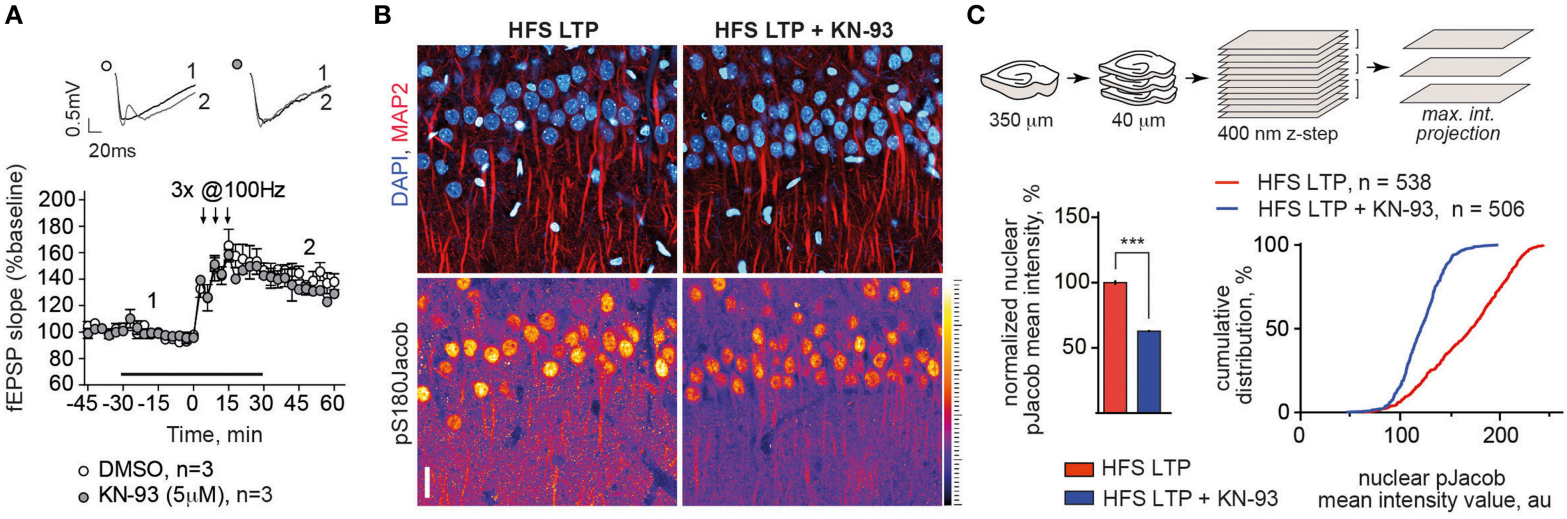
**Blocking of CaMKII-α activity during LTP induction with HFS reduced the amount of pJacob in the nuclei of CA1 pyramidal neurons. (A)** KN-93 in concentration of 5 μM has no effect on late-LTP induction using HFS. Graphs show normalized to baseline fEPSP slope values. Insets show examples of fEPSP slopes taken at the time point indicated. **(B)** Incubation of acute hippocampal slices with KN-93 during LTP induction significantly reduces nuclear pJacob IR. Depicted are representative maximum intensity projection (MIP) confocal images from 4 optical sections (400 nm z-step size) of rat hippocampal slices incubated ether with KN-93 or with DMSO control during LTP induction and immunolabeled against pS180Jacob. Scale bar is 20 μm. **(C)** Sketch illustrates procedure of image acquisition and processing, upper panel. Graphs show averaged normalized nuclear pJacob mean intensities with and without KN-93 during LTP induction as well as cumulative frequency distribution that represents population of pS180Jacob positive CA1 nuclei with and without KN-93 in a.u. Relative fluorescence intensities of pS180Jacob IR after LTP induction in CA1 region of treated with KN-93 hippocampal slices were normalized to untreated control, ^***^p < 0.001; one-way ANOVA followed by Bonferroni *post-hoc* test.

In previous work we could also show that induction of NMDAR-dependent LTP following high-frequency stimulation of Schaffer collaterals results in increased nuclear import of Jacob phosphorylated at Serine180 as detected with staining of an anti-pS180-Jacob (Karpova et al., 2013). We repeated this experiment and performed immunohistochemical stainings under identical conditions (Figures 5A–C). IHC staining utilizing the phospho antibody confirmed the findings above as we found that KN-93 application prevented nuclear import of pJacob (Figures 5B,C) inasmuch without affecting the induction of LTP (Figure 5A). This is evidenced by the normalized nuclear pJacob mean intensity and a shift in the cumulative distribution of the mean intensity value (Figures 5B,C). Thus, nuclear translocation of ERK and Jacob in response to stimuli that induce NMDAR-dependent LTP is sensitive to CaMKII-α inhibiton.

### Nuclear transport of pERK/pJacob in mature hippocampal neurons does not depend on Ras-GEF activity of RasGRF2

Hippocampal primary neurons at DIV16 express already relatively high levels of GluN2A but very low levels of RasGRF2, whose expression increases later in development (Tian et al., 2004; Li et al., 2006). Previous reports indicate a prominent role of RasGRF2 in control synaptic ERK-activity in mature neurons (Fernandez-Medarde and Santos, 2011). To investigate a potential contribution of RasGRF2 in activity-dependent nuclear transport of ERK and Jacob we employed a shRNA (shRNA1) construct that specifically knocked down protein expression of RasGRF2 (Figures S2A,B) but not those of the closely related RasGRF1 (Figure S2C). Moreover, RasGRF1 is barely expressed in hippocampal primary neurons because of imprinting of the gene in primary cultures (Schwechter et al., 2013).

In the first set of experiments we found that viral infection of cortical primary neurons (DIV17 and DIV21) with a RasGRF2 shRNA1 had no effect on basal pERK levels in accord with our previous observation (Bido et al., 2015), but it reduced the 4-AP/bicuculline induced increase of pERK in immunoblot experiments in mature cultures (Figure S2D).

**Figure 6:**
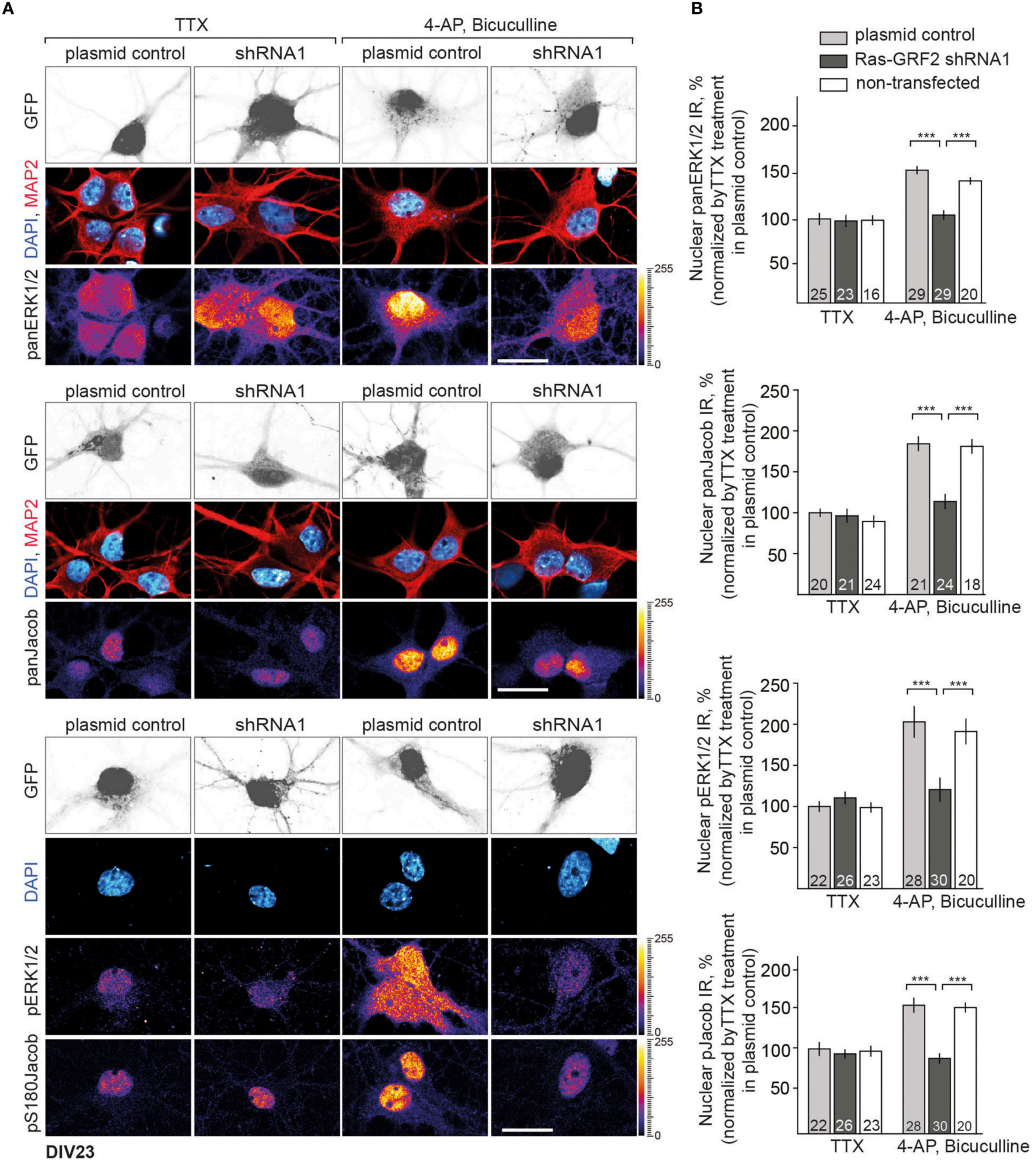
**Depletion of RasGRF2 negatively affects Jacob/ERK nuclear signaling. (A,B)** Nuclear trafficking of panERK/pERK and panJacob/pJacob following NMDAR activation in RasGRF2 knocked down hippocampal neurons. Scale bar is 20 μm, ^***^p < 0.001; one-way ANOVA followed by Bonferroni *post-hoc* test.

**Figure 7:**
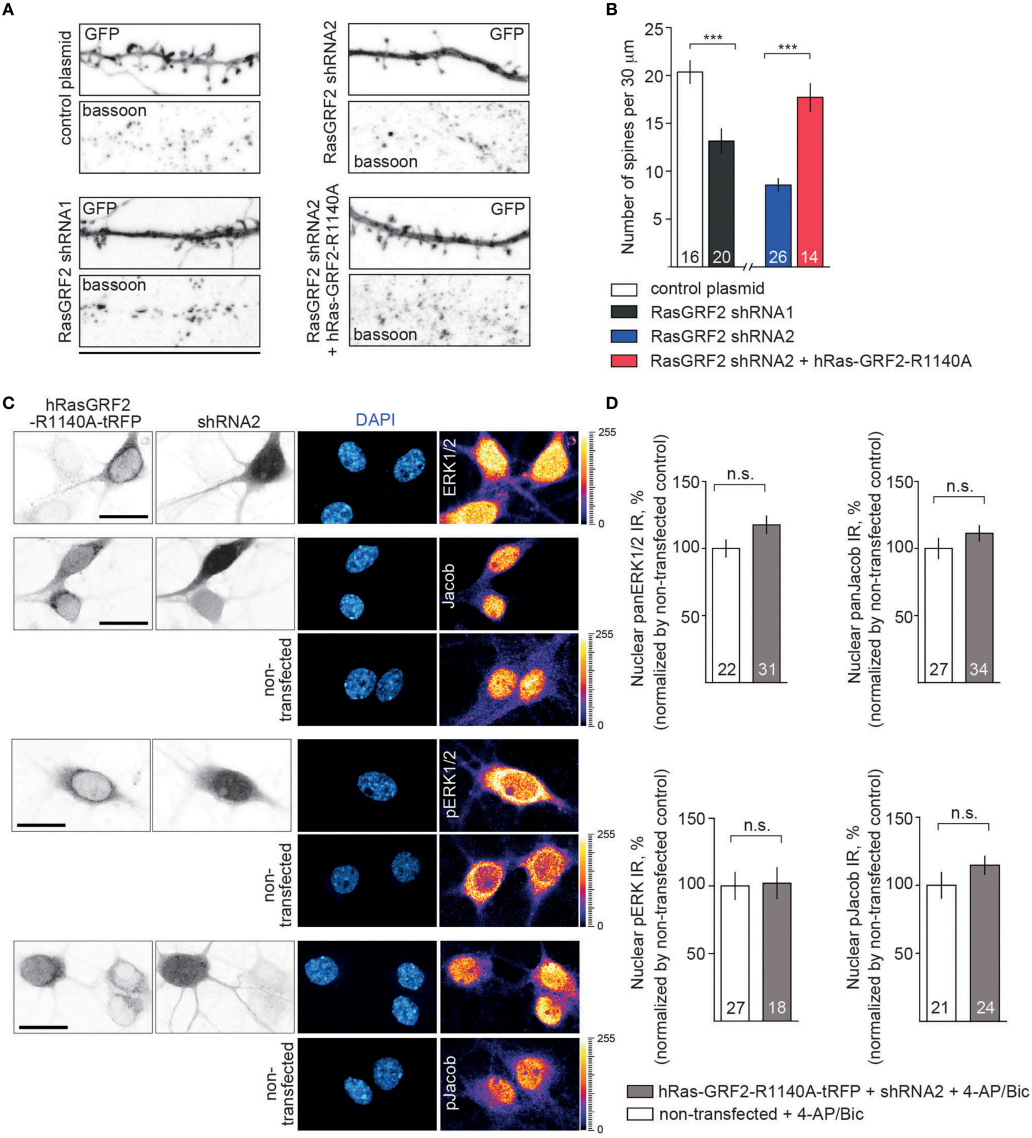
**Depletion of RasGRF2 negatively affects spine number in Rac1-dependent manner and the effects of Jacob/ERK nuclear signaling upon enhanced synaptic activity can be rescued by expressing of shRNA resistant hRasGRF2 rescuing Rac-GEF, but not Ras-GEF activity. (A,B)** shRNA1 mediated RasGRF2 KD resulted in decreased spine density. Molecular replacement of ratRasGRF2 with hRasGRF2 lacking only Ras-GEF function (RasGEF-dead-R1140A-RasGRF2) prevented the deleterious effects of knock-down of the rat protein on spine number and morphology. Scale bar is 25 μm, ^***^p < 0.001; one-way ANOVA followed by Bonferroni *post-hoc* test. **(C,D)** Nuclear trafficking of panERK/pERK and panJacob/pJacob following stimulation of synaptic NMDAR in conditions of rat RasGRF2 down-regulation and overexpression of human shRNA resistant RasGEF-dead-R1140A-RasGRF2-tRFP has no differences from non-transfecting neurons. Scale bar is 20 μm, n.s., is non-significant by one-way ANOVA followed by Bonferroni *post-hoc* test.

We next used this shRNA1 construct for plasmid transfection of hippocampal primary neurons at DIV16 and DIV23 (Figure S3 and Figure 6). A RasGRF2 protein knockdown had no effect on the activity-dependent nuclear import of panJacob/pJacob, panERK/pERK at DIV16 (Figure S3), a time point when RasGFR2 is barely detectable in hippocampal primary neurons (Li et al., 2006). In stark contrast we found that transfection of this construct significantly reduced nuclear ERK, pERK, Jacob, and pJacob immunofluorescence when we transfected neurons at DIV17 and performed the stimulation experiment at DIV23. Basal nuclear immunofluorescence levels of ERK and Jacob were not affected (Figure 6). This finding was surprising. However, we realized that, in accord to Schwechter et al. (2013), a RasGRF2 shRNA knockdown in older hippocampal primary neurons (DIV23) resulted in a clearly reduced number of spines (Figures 7A,B). We therefore reasoned that structural defects in spine morphology and in consequence reduced synaptic strength might underlie the reduced phosphorylation of ERK and nuclear import of Jacob and ERK. RasGRF2 has dual GEF activities for Rac1 and Ras and it was shown that its Rac-GEF activity is important for spinous actin dynamics and that Rac-GEF activity of RasGRF2 controls synapse number and spine volume (Schwechter et al., 2013). To distinguish the dual signaling properties of RasGRF2 and their effect on ERK phosphorylation we employed a previously established molecular replacement strategy (Schwechter et al., 2013). In order to rescue only Rac-GEF, but not Ras-GEF activity of RasGRF2, endogenous rat RasGRF2 was replaced with shRNA resistant human RasGRF2 (shRNA2, Figure S2A). Thus, we knocked down rat RasGRF2 in primary neurons at DIV17 and in parallel re-expressed RNAi-resistant human RasGRF2 carrying an inactivating mutation in the catalytic Ras-GEF domain (RasGEF-dead-R1140A-RasGRF2) whereas the Rac-GEF activity was not affected in this mutant. As reported previously (Schwechter et al., 2013), re-expression of the human Ras-GEF-dead mutant of RasGRF2 prevented the deleterious effects of knockdown of the rat protein on spine number (Figures 7A,B). Most important, replacement of the rat by the Ras-GEF-dead mutant human protein also prevented the effects of knockdown of the endogenous protein on nuclear trafficking of phosphorylated ERK and Jacob following stimulation of synaptic NMDAR (Figures 7C,D).

Thus, restoration of structural defects by re-expressing RasGRF2 with a functional Rac-GEF but inactive Ras-GEF domain is sufficient to restore activity-dependent phosphorylation and nuclear transport of ERK and Jacob.

## Discussion

In this paper we have addressed the issue how synaptic NMDAR-signals are coupled to phosphorylation and subsequent nuclear import of pERK1/2 and pJacob. We could demonstrate that CaMKII-α is downstream of GluN2B containing NMDAR whose activation results in nuclear accumulation of pERK/pJacob. The data suggest that in mature hippocampal and cortical pyramidal neurons pERK/pJacob translocate together to the nucleus in a GluN2B/CamKII-α dependent manner. Along these lines, co-immunoprecipitation experiments suggest that GluN2B, CaMKII-α, ERK, and Jacob might be in one complex *in vivo* (Karpova et al., 2013; Dinamarca et al., 2016). Most important, we found that inhibition of CaMKII-α interrupted the nuclear import of ERK as well as those of Jacob and that synaptic GluN2B containing NMDAR are instrumental for the nuclear accumulation of pERK at least at later time points (i.e., ≈ 30 min) following stimulation. Previous work and the present study suggest that import of ERK at later time points relies to a large degree on binding of ERK to Jacob and active transport from synapse-to-nucleus (Karpova et al., 2013; Spilker et al., 2016). Induction of LTP in distant dendritic branches elicits nuclear responses which are delayed by tens of minutes, in correlation with the distance of the stimulation, suggesting that propagation was via a relatively slow mechanism (Zhai et al., 2013). Nuclear ERK activity is enhanced within the same time window required for Jacob nuclear import raising the possibility that both proteins translocate together to the nucleus (Zhai et al., 2013). Taken together the data make it plausible that the ERK/Jacob signalosome for long-distance transport might be already assembled at synaptic GluN2B-containing NMDAR complexes. Whether CaMKII-α itself is part of the ERK/Jacob signalosome is at present unclear. Of note, a recent study showed nuclear translocation of CaMKII-γ (Ma et al., 2014), a translocation, however, that was initiated by a Ca^2+^-signal of L-type voltage-dependent Ca^2+^-channels and nuclear translocation was probably not based on active transport.

A number of questions arise from these findings. RasGRF2 is reportedly coupled to GluN2A-containing NMDAR and activated by Ca^2+^/CaM and thus upstream of Ras-mediated synaptic activation of ERK. RasGRF2 signaling has been linked to synaptic plasticity and maintenance of LTP (Li et al., 2006). We observed that, like CaMKII-α inhibition, a RasGRF2 shRNA knockdown also significantly attenuated nuclear accumulation of pERK/pJacob following 4-AP/bicuculline treatment in primary neurons at DIV23. Subsequent analysis, however, revealed that a protein knockdown in mature neurons resulted in spine loss, a finding that has been reported by Schwechter et al. (2013). It was shown previously that the spine phenotype induced by shRNA knockdown of RasGRF2 is largely due to the RacGEF and not the RasGEF activity of the protein (Schwechter et al., 2013). To distinguish between the possibilities that a structural defect or lack of RasGEF signaling is responsible for the lack of nuclear transport we next re-expressed a shRNA resistant Ras-dead mutant of RasGRF2 following shRNA knockdown of the endogenous protein. In accordance with Schwechter et al. (2013) we found a rescue of the spine phenotype and despite the lack of RasGEF activity we observed nuclear translocation of both pERK and pJacob in response to enhanced synaptic activity. Taken together the data indicate that Ras-signaling downstream of RasGRF2 is not essential for long-distance transport of pJacob/pERK to the nucleus.

Induction of NMDAR-dependent LTP at hippocampal synapses can trigger Ras-signaling and subsequent activation of ERK *via* both CaMKII-α and RasGRF2. However, only CaMKII-α triggers ERK-dependent S180 phosphorylation of Jacob and nuclear translocation of pERK/pJacob. This might be explained by the existence of different complexes at different NMDAR subtypes. In this and previous studies we found that Jacob primarily associates with GluN2B containing NMDAR, whereas work of others shows that RasGRF2 might preferentially associate with NMDAR containing GluN2A. Nonetheless published work suggests that a large fraction of synaptic NMDAR contains both subunits (Rauner and Köhr, 2011) and it is therefore unclear how ERK-signaling is regulated downstream of these receptors. Docking to the cytoplasmic tail of GluN2B might constitute a nanodomain for phosphorylation of ERK and Jacob. Another issue is the relatively late expression of RasGRF2 in hippocampal neurons, which might obscure a role of the protein in cell culture experiments. To address this issue we also performed experiments in acute hippocampal slices where we could block nuclear transport of pERK/pJacob with a CaMKII-α antagonist after the induction of LTP. Thus, it is unlikely that the maturation of neurons or culture conditions can account for the findings. In summary, the present study delineates a signaling pathway from synaptic GluN2B NMDAR that essentially requires CaMKII-α for the nuclear transport of pERK/pJacob that is likely to be important for CREB activation and plasticity-related gene expression. This does not exclude the possibility that other pathways might exist that rely on synaptic GluN2A/RasGRF2-signaling to the nucleus. Of note a recent paper identified RNF10 as a GluN2A specific synapse-to-nucleus protein messenger (Dinamarca et al., 2016). A potential role of RasGRF2 and pERK for translocation was not investigated in this study but it is plausible that RasGRF2 might trigger nuclear trafficking of proteins other than Jacob from synapses undergoing NMDAR-dependent LTP.

## Author contributions

AK and MK designed the research. MM performed stimulation experiments with primary neurons and cLTP in hippocampal slices followed nuclear isolation, as well as characterized the shRNA KD; AK performed molecular cloning, IHC and co-immunoprecipitations experiments; PY contributed to electrophysiology, RB contributed with virus production. AK and MM contributed to analysis, AK and MK wrote the manuscript.

## Funding

This work was supported by: DFG (Kr1879/5-1, 6-1 to MRK; SFB779 TPB8 to AK and MK); JPND STAD to MK; Federal State of Saxony-Anhalt, Center for Behavioral Brain Sciences (CBBS, NeuroNetwork #5) to AK; Fondazione Mariani for Neurological Disorders, the Michael J. Fox Foundation for Parkinson's Research, Parkinson's UK, the Italian Ministry of Health and CARIPLO Foundation to RB.

### Conflict of interest statement

The authors declare that the research was conducted in the absence of any commercial or financial relationships that could be construed as a potential conflict of interest.

## References

[B1] Behnisch T. Yuanxiang P. Bethge P. Parvez S. Chen Y. Yu J. (2011). Nuclear translocation of Jacob in hippocampal neurons after stimuli inducing long-term potentiation but not long-term depression. PLoS ONE 6:e17276 10.1371/journal.pone.0017276 21364755 PMC3041791

[B2] Bido S. Solari N. Indrigo M. D'Antoni A. Brambilla R. Morari M. (2015). Differential involvement of Ras-GRF1 and Ras-GRF2 in L-DOPA-induced dyskinesia. Ann. Clin. Transl. Neurol. 6, 662–678. 10.1002/acn3.202 26125041 PMC4479526

[B3] Boehm J. Kang M. G. Johnson R. C. Esteban J. Huganir R. L. Malinow R. (2006). Synaptic incorporation of AMPA receptors during LTP is controlled by a PKC phosphorylation site on GluR1. Neuron 51, 213–225. 10.1016/j.neuron.2006.06.013 16846856

[B4] Brambilla R. Gnesutta N. Minichiello L. White G. Roylance A. J. Herron C. E.. (1997). A role for the Ras signalling pathway in synaptic transmission and long-term memory. Nature 390, 281–286. 10.1038/36849 9384379

[B5] Cai F. Frey J. U. Sanna P. P. Behnisch T. (2010). Protein degradation by the proteasome is required for synaptic tagging and the heterosynaptic stabilization of hippocampal late-phase long-term potentiation. Neuroscience 169, 1520–1526. 10.1016/j.neuroscience.2010.06.032 20600658

[B6] Chandler L. J. Sutton G. Dorairaj N. R. Norwood D. (2001). N-methyl D-aspartate receptor-mediated bidirectional control of extracellular signal-regulated kinase activity in cortical neuronal cultures. J. Biol. Chem. 276, 2627–2636. 10.1074/jbc.M003390200 11062237

[B7] Chen Q. He S. Hu X. L. Yu J. Zhou Y. Zheng J.. (2007). Differential roles of NR2A- and NR2B-containing NMDA receptors in activity-dependent brain-derived neurotrophic factor gene regulation and limbic epileptogenesis. J. Neurosci. 27, 542–552. 10.1523/JNEUROSCI.3607-06.2007 17234586 PMC6672795

[B8] Dieterich D. C. Karpova A. Mikhaylova M. Zdobnova I. König I. Landwehr M.. (2008). Caldendrin-Jacob: a protein liaison that couples NMDA receptor signalling to the nucleus. PLoS Biol. 6:e34. 10.1371/journal.pbio.0060034 18303947 PMC2253627

[B9] Dinamarca M. C. Guzzetti F. Karpova A. Lim D. Mitro N. Musardo S.. (2016). Ring finger protein 10 is a novel synaptonuclear messenger encoding activation of NMDA receptors in hippocampus. Elife 5:e12430. 10.7554/eLife.12430 26977767 PMC4805553

[B10] El Gaamouch F. Buisson A. Moustié O. Lemieux M. Labrecque S. Bontempi B.. (2012). Interaction between αCaMKII and GluN2B controls ERK-dependent plasticity. J. Neurosci. 32, 10767–10779. 10.1523/JNEUROSCI.5622-11.2012 22855824 PMC6621385

[B11] Fasano S. Brambilla R. (2011). Ras-ERK signaling in behaviour: old questions and new perspectives. Front. Behav. Neurosci. 5:79 10.3389/fnbeh.2011.00079 PMC3223382 22131969

[B12] Fernandez-Medarde A. Santos E. (2011). The RasGrf family of mammalian guanine nucleotide exchange factors. Biochim. Biophys. Acta 1815, 170–188. 10.1016/j.bbcan.2010.11.001 21111786

[B13] Foster K. A. McLaughlin N. Edbauer D. Phillips M. Bolton A. Constantine-Paton M.. (2010). Distinct roles of NR2A and NR2B cytoplasmic tails in long-term potentiation. J. Neurosci. 30, 2676–2685. 10.1523/JNEUROSCI.4022-09.2010 20164351 PMC2840640

[B14] Gao C. Gill M. B. Tronson N. C. Guedea A. L. Guzmán Y. F. Huh K. H.. (2010). Hippocampal NMDA receptor subunits differentially regulate fear memory formation and neuronal signal propagation. Hippocampus 20, 1072–1082. 10.1002/hipo.20705 19806658 PMC2891656

[B15] Gomes G. M. Dalmolin G. D. Bär J. Karpova A. Mello C. F. Kreutz M. R. (2014). Alterations in the polyamine system underlie memory impairment and synaptic dysfunction induced by amyloid β-peptide25-35. PLoSONE 9:e99184 10.1371/journal.pone.0099184 PMC4055672 24921942

[B16] Jang S. S. Royston S. E. Xu J. Cavaretta J. P. Vest M. O. Lee K. Y.. (2015). Regulation of STEP_61_ and tyrosine-phosphorylation of NMDA and AMPA receptors during homeostatic synaptic plasticity. Mol. Brain 8, 55. 10.1186/s13041-015-0148-4 26391783 PMC4578242

[B17] Jin S. X. Feig L. A. (2010). Long-term potentiation in the CA1 hippocampus induced by NR2A subunit-containing NMDA glutamate receptors is mediated by Ras-GRF2/Erk map kinase signaling. PLoS ONE 5:e11732. 10.1371/journal.pone.0011732 20661302 PMC2908693

[B18] Jordan B. A. Kreutz M. R. (2009). Nucleocytoplasmic protein shuttling: the direct route in synapse-to-nucleus signaling. Trends Neurosci. 32, 392–401. 10.1016/j.tins.2009.04.001 19524307

[B19] Kapitein L. C. Schlager M. A. van der Zwan W. A. Wulf P. S. Keijzer N. Hoogenraad C. C. (2010). Probing intracellular motor protein activity using an inducible cargo trafficking assay. Biophys. J. 99, 2143–2152. 10.1016/j.bpj.2010.07.055 20923648 PMC3042561

[B20] Karpova A. Bär J. Kreutz M. R. (2012). Long-distance signaling from synapse to nucleus via protein messengers. Adv. Exp. Med. Biol. 970, 355–376. 10.1007/978-3-7091-0932-8_16 22351064

[B21] Karpova A. Mikhaylova M. Bera S. Bär J. Reddy P. P. Behnisch T.. (2013). Encoding and transducing the synaptic or extrasynaptic origin of NMDA receptor signals to the nucleus. Cell 152, 1119–1133. 10.1016/j.cell.2013.02.002 23452857

[B22] Karpova A. Mikhaylova M. Thomas U. Knöpfel T. Behnisch T. (2006). Involvement of protein synthesis and degradation in long-term potentiation of Schaffer collateral CA1 synapses. J. Neurosci. 26, 4949–4955. 10.1523/JNEUROSCI.4573-05.2006 16672670 PMC6674165

[B23] Kaushik R. Grochowska K. M. Butnaru I. Kreutz M. R. (2014). Protein trafficking from synapse-to-nucleus in control of activity-dependent gene expression. Neuroscience 280, 340–350. 10.1016/j.neuroscience.2014.09.011 25230285

[B24] Kim M. J. Dunah A. W. Wang Y. T. Sheng M. (2005). Differential roles of NR2A-and NR2B-containing NMDA receptors in Ras-ERK signaling and AMPA receptor trafficking. Neuron 46, 745–760. 10.1016/j.neuron.2005.04.031 15924861

[B25] Kindler S. Dieterich D. C. Schütt J. Sahin J. Karpova A. Mikhaylova M. (2009). Dendritic mRNA targeting of Jacob and NMDA-induced nuclear translocation pool after Calpain-mediated proteolysis. J. Biol. Chem. 284, 25431–25440. 10.1074/jbc.M109.022137 19608740 PMC2757244

[B26] Krapivinsky G. Krapivinsky L. Manasian Y. Ivanov A. Tyzio R. Pellegrino C.. (2003). The NMDA receptor is coupled to the ERK pathway by a direct interaction between NR2B and RasGRF1. Neuron 40, 775–784. 10.1016/S0896-6273(3)00657-7 14622581

[B27] Li S. Tian X. Hartley D. M. Feig L. A. (2006). Distinct roles for ras-guanine nucleotide-releasing factor-1 (Ras-GRF1) and Ras-GRF2 in the induction of long-term potentiation and long-term depression. J. Neurosci. 26, 1721–1729. 10.1523/JNEUROSCI.3990-05.2006 16467520 PMC6793631

[B28] Ma H. Groth R. D. Cohen S. M. Emery J. F. Li B. Hoedt E. (2014). γCaMKII shuttles Ca^2+^/CaM to the nucleus to trigger CREB phosphorylation and gene expression. Cell 159, 281–294. 10.1016/j.cell.2014.09.019 25303525 PMC4201038

[B29] Mikhaylova M. Karpova A. Bär J. Behnisch T. Zuschratter W. Kreutz M. R. (2014). Cellular distribution of the synapto-nuclear protein messenger Jacob in the rat brain. Brain Struct. Funct. 219, 843–860. 10.1007/s00429-013-0539-1 23539133

[B30] Mulholland P. J. Carpenter-Hyland E. P. Hearing M. C. Becker H. C. Woodwald J. J. Chandler L. J. (2008). Glutamate transporters regulate extrasynaptic NMDA receptor modulation of Kv2.1 potassium channels. J. Neurosci. 28, 8801–8809. 10.1523/JNEUROSCI.2405-08.2008 18753382 PMC2612131

[B31] Otmakhov N. Khibnik L. Otmakhova N. Carpenter S. Riahi S. Asrican B.. (2004). Forskolin-induced LTP in the CA1 hippocampal region is NMDA receptor dependent. J. Neurophysiol. 91, 1955–1962. 10.1152/jn.00941.2003 14702333

[B32] Panayotis N. Karpova A. Kreutz M. R. Fainzilber M. (2015). Macromolecular transport in synapse to nucleus communication. Trends Neurosci. 38, 108–116. 10.1016/j.tins.2014.12.001 25534890

[B33] Paoletti P. Bellone C. Zhou Q. (2013). NMDA receptor subunit diversity: impact on receptor properties, synaptic plasticity and disease. Nat. Rev. Neurosci. 14, 383–400. 10.1038/nrn3504 23686171

[B34] Proepper C. Johannsen S. Liebau S. Dahl J. Vaida B. Bockmann J.. (2007). Abelson interacting protein 1 (Abi-1) is essential for dendrite morphogenesis and synapse formation. EMBO J. 26, 1397–1409. 10.1038/sj.emboj.7601569 17304222 PMC1817621

[B35] Rauner C. Köhr G. (2011). Triheteromeric NR1/NR2A/NR2B receptors constitute the major N-methyl-D-aspartate receptor population in adult hippocampal synapses. J. Biol. Chem. 286, 7558–7566. 10.1074/jbc.M110.182600 21190942 PMC3045010

[B36] Redondo R. L. Okuno H. Spooner P. A. Frenguelli B. G. Bito H. Morris R. G. M. (2010). Synaptic tagging and capture: differential role of distinct calcium/calmodulin kinases in protein synthesis-dependent long-term potentiatio. J. Neurisci. 30, 4981– 4989. 10.1523/JNEUROSCI.3140-09.2010 20371818 PMC6632790

[B37] Rönicke R. Mikhaylova M. Rönicke S. Meinhardt J. Schröder U. H. Fändrich M.. (2011). Early neuronal dysfunction by amyloid beta oligomers depends on activation of NR2B-containing NMDA receptors. Neurobiol. Aging 32, 2219–2228. 10.1016/j.neurobiolaging.2010.01.011 20133015

[B38] Schwechter B. Rosenmund C. Tolias K. F. (2013). RasGRF2 Rac-GEF activity couples NMDA receptor calcium flux to enhanced synaptic transmission. Proc. Natl. Acad. Sci. U.S.A. 110, 14462–14467. 10.1073/pnas.1304340110 23940355 PMC3761609

[B39] Shaomin L. I. Xuejun T. M. H. Feig L. A. (2006). Distinct roles for ras-guanine nucleotide releasing factor 1 (Ras-GRF1) and Ras-GRF2 in the induction of long-term potentiation and long-term depression. J. Neurosci. 26, 1721–1729. 10.1523/JNEUROSCI.3990-05.2006 16467520 PMC6793631

[B40] Shen K. Teruel M. N. Subramanian K. Meyer T. (1998). CaMKIIbeta functions as an F-actin targeting module that localizes CaMKIIalpha/beta heterooligomers to dendritic spines. Neuron 21, 593–606. 10.1016/S0896-6273(00)80569-3 9768845

[B41] Spilker C. Nullmeier S. Crochowska K. M. Schumacher A. Butnatu I. Macharadze T.. (2016). A Jacob/Nsmf gene knockout results in hippocampal dysplasia and impaired BDNF signaling in dendritogenesis. PLoS Genet. 12:e1005907. 10.1371/journal.pgen.1005907 26977770 PMC4792503

[B42] Sutton G. Chandler L. J. (2002). Activity-dependent NMDA receptor-mediated activation of protein kinase B/Akt in cortical neuronal cultures. J. Neurochem. 82, 1097–1105. 10.1046/j.1471-4159.2002.01031 12358757

[B43] Tian X. Gotoh T. Tsuji K. Lo E. H. Huang S. Feig L. A. (2004). Developmentally regulated role for Ras-GRFs in coupling NMDA glutamate receptors to Ras, Erk and CREB. EMBO J. 23, 1567–1575. 10.1038/sj.emboj.7600151 15029245 PMC391062

[B44] Yuanxiang P. Bera S. Karpova A. Kreutz M. R. Mikhaylova M. (2014). Isolation of CA1 nuclear enriched fractions from hippocampal slices to study activity dependent nuclear import of synapto-nuclear messenger proteins. J. Vis. Exp. e51310. 10.3791/51310 25145907 PMC4758714

[B45] Zhai S. Ark E. D. Parra-Buenco P. Yasuda R. (2013). Long-distance integration of nuclear ERK signaling triggered by activation of a few dendritic spines. Science 342, 1107–1111. 10.1126/science.1245622 24288335 PMC4318497

