# Supplementary material for: Synaptic GluN2B/CaMKII-α Signaling Induces Synapto-Nuclear Transport of ERK and Jacob
Source: Front Mol Neurosci. 2016 Aug 10;9:66. doi: 10.3389/fnmol.2016.00066 (PMC4978723; doi:10.3389/fnmol.2016.00066)
Supplement: Supplementary file 1 [file DataSheet1.DOCX]

Supplementary Material

Synaptic GluN2B / CaMKII-α signalling induces synapto-nuclear transport of ERK and Jacob

**Michelle Melgarejo da Rosa^1^, PingAn Yuanxiang^1^, Riccardo Brambilla^2^, Michael R. Kreutz^1,3^, Anna Karpova^1*^**

*** Correspondence:** Anna Karpova, Karpova@lin-magdeburg.de

# Supplementary Figures


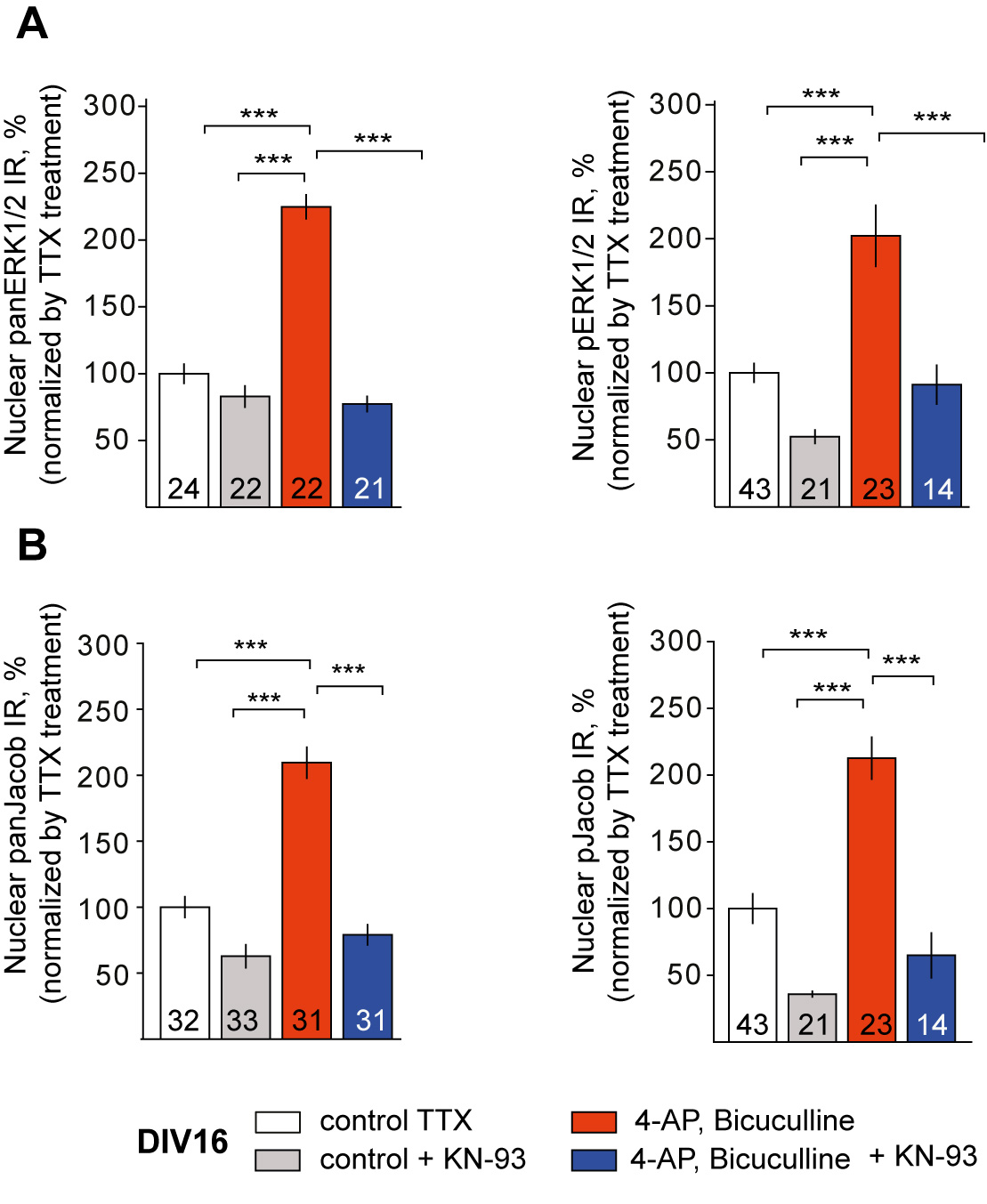


**Supplementary Figure 1.** CaMKII-α activity controls nuclear import of ERK/pERK **(A)** and Jacob/pJacob **(B)** in hippocampal neurons at DIV16. ***p<0.001; one-way ANOVA followed by Bonferroni post-hoc test.


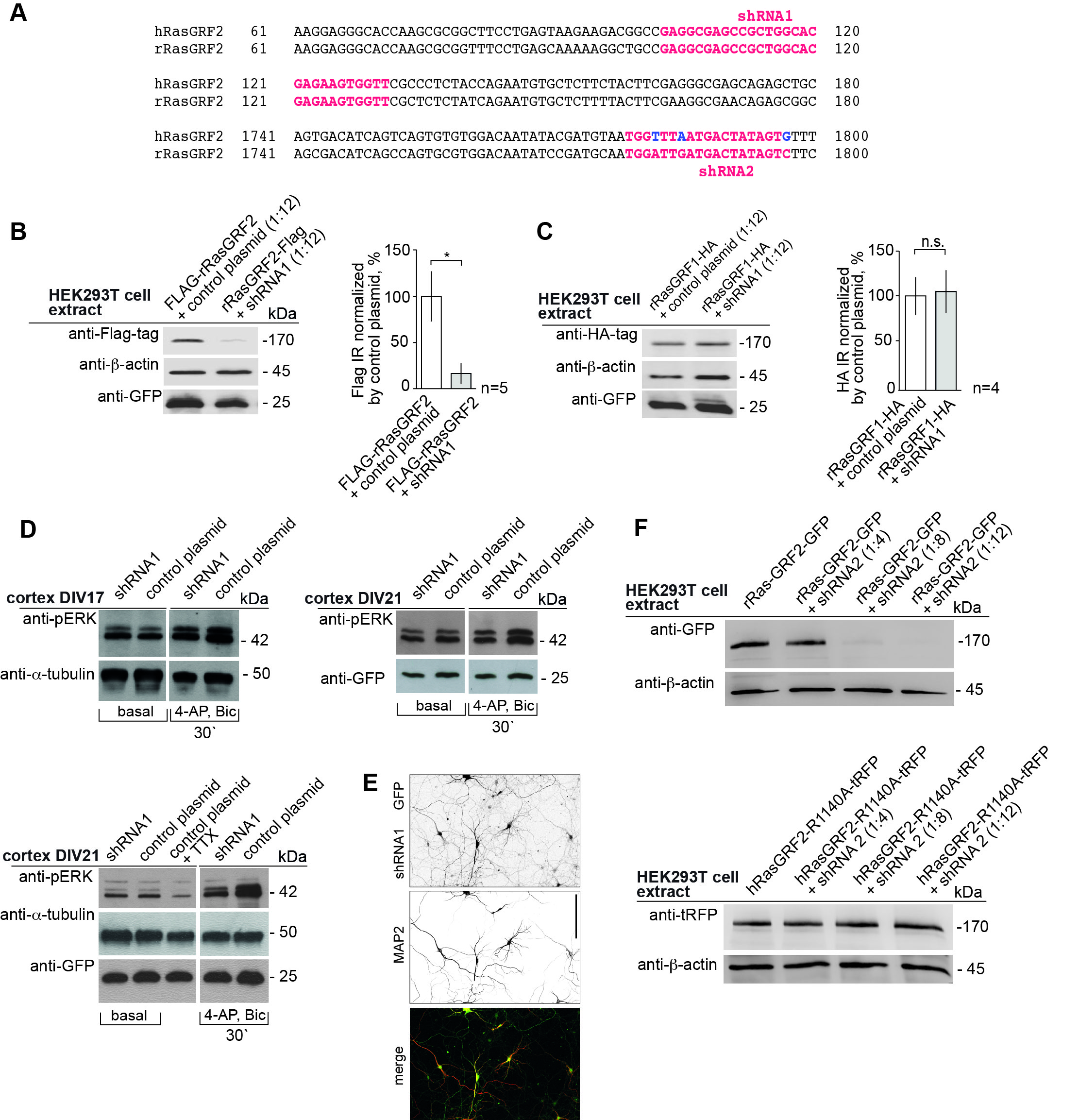


**Supplementary Figure 2.** Selectivity and efficiency of shRNA knockdown of rat RasGRF2.

**(A)** Sequence alignment of human and rat RasGRF2 related to shRNA targeting sequences (shRNA1 and shRNA2, indicated in red) accordingly. **(B)** Efficiency of rat RasGRF2 KD confirmed in heterologous system by co-expressing FLAG-target rat RasGRF2 together with shRNA1. **(C)** shRNA1 selectively down-regulates rat RasGRF2 but not rat RasGRF1. **(D)** Viral transduction of cortical neurons with shRNA1 does not alter basal pERK level but robustly attenuates an increase in pERK IR during enhanced synaptic activity in mature neurons. **(E)** Depicted are confocal images of cortical primary neurons DIV23 representing lenti-viral transduction efficiency for RasGRF2 shRNA1 where all MAP2 positive cells express GFP. Scale bar is 200 μm. **(F)** Depicted are WBs confirming that shRNA2 down-regulates ratRasGRF2 fused to GFP and not the hRasGRF2-R1140A-tagRFP in heterologous system.

**
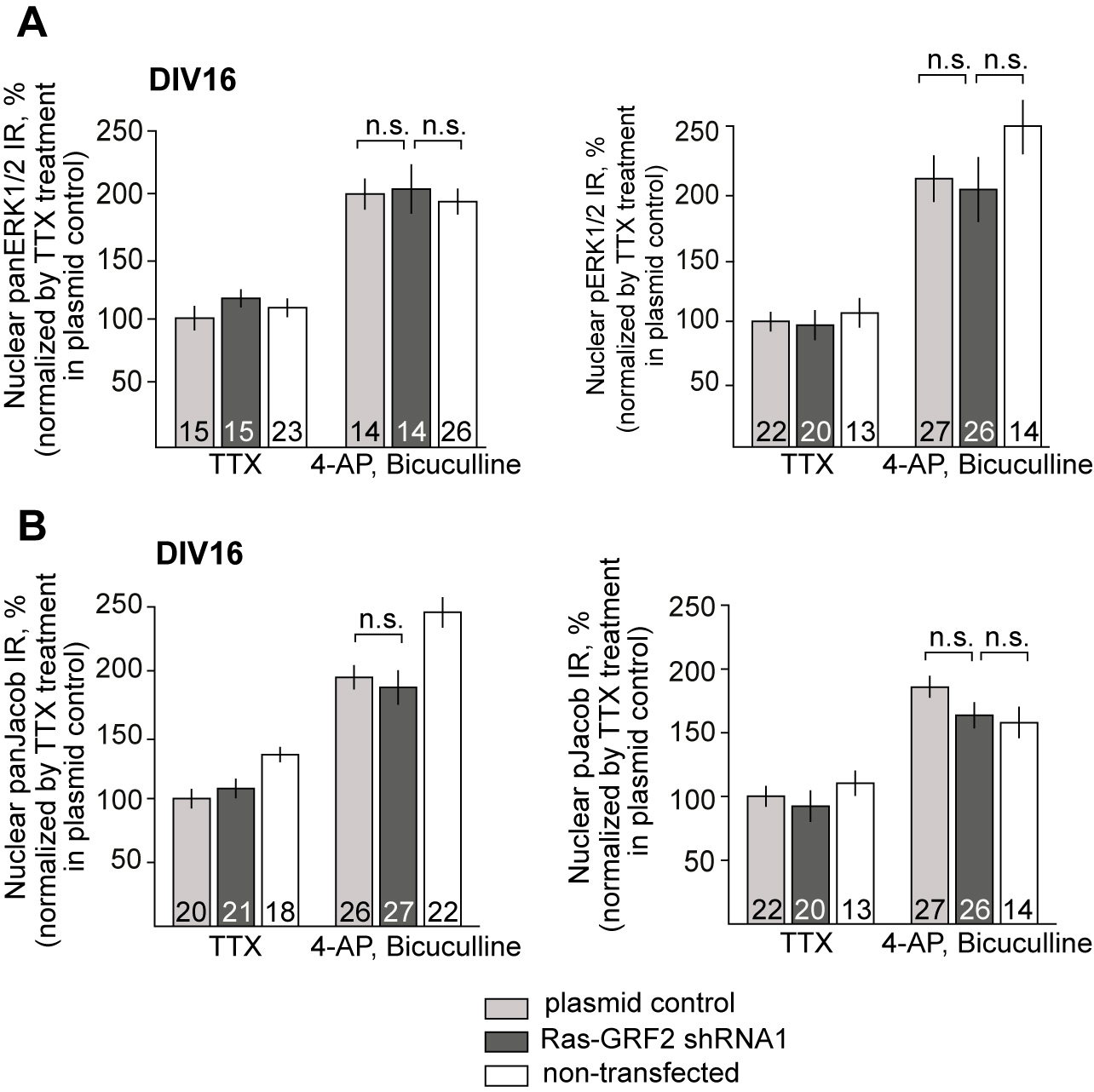
**

**Supplementary Figure 3.** A RasGRF2 protein knockdown has no effect on the activity-dependent nuclear import of panERK/pERK **(A)** and panJacob/pJacob **(B)** at DIV16; n.s. by one-way ANOVA followed by Bonferroni post-hoc test.
